# Sex-driven factors associated with anxiety and depression in autoimmune diabetes

**DOI:** 10.1007/s00592-024-02275-4

**Published:** 2024-05-14

**Authors:** Enrico Saudelli, Simona Moscatiello, Michele Baldari, Claudio Bongiorno, Stefano Zucchini, Giulio Maltoni, Alessandro Agostini, Alexandro Paccapelo, Elena Nardi, Danilo Ribichini, Alessia Bruco, Valentina Lo Preiato, Gilberto Laffi, Uberto Pagotto, Guido Di Dalmazi

**Affiliations:** 1 Division of Endocrinology and Diabetes Prevention and Care, IRCCS Azienda Ospedaliero-Universitaria di Bologna, Bologna, Italy; 2 Department of Medical and Surgical Sciences (DIMEC), Alma Mater Studiorum University of Bologna, Bologna, Italy; 3 Pediatric Unit, IRCCS Azienda Ospedaliero-Universitaria di Bologna, Bologna, Italy; 4 Research and Innovation Unit, IRCCS Azienda Ospedaliero-Universitaria di Bologna, Bologna, Italy

**Keywords:** Type 1 diabetes mellitus, Autoimmune diabetes, Hospital Anxiety and Depression Scale, HADS, Continuous glucose monitoring

## Abstract

**Aim:**

To analyze the prevalence of anxiety and depression in a large cohort of adults with autoimmune diabetes, identifying sex-driven associated factors.

**Materials and methods:**

In this cross-sectional study, we enrolled 553 consecutive adults with Type 1 diabetes mellitus or latent autoimmune diabetes in adults who came to the Division of Endocrinology of the S.Orsola-Malpighi Polyclinic, Bologna (Italy), to receive their second dose of SARS-CoV-2 vaccine. We administered the questionnaires: Hospital Anxiety and Depression Scale, Diabetes Distress Scale, Diabetes-related Quality of Life, Diabetes Treatment Satisfaction Questionnaire. We collected clinical and biochemical data and 14 days glucose metrics in patients with sensor use > 70% in a time span of ± 4 months from the questionnaires’ administration. We excluded 119 patients from our analyses with missing data (final cohort *n* = 434: 79% of those enrolled).

**Results:**

Anxiety and depression prevalence was respectively 30.4% and 10.8%. According to the multivariate analysis, higher diabete-related emotional burden, lower treatment satisfaction, but not physician-related distress, were risk factors for anxiety and depression; female sex was associated with anxiety (OR 0.51, 95% 0.31–0.81; *p* = 0.005); in women, depression was associated with increasing age (males vs. females OR 0.96 per 1 year increase, 95% CI 0.92–1.00; *p* = 0.036), whilst in men with HbA1c (OR 1.08 per 1 mmol/mol increase, 95% CI 1.03–1.13; *p* = 0.002).

**Conclusion:**

Nearly 1/3 of patients with autoimmune diabetes suffers from anxiety and 1/10 from depression. These conditions are associated with independent modifiable and non-modifiable characteristics. For depression, these characteristics differ between males and females.

**Supplementary Information:**

The online version contains supplementary material available at 10.1007/s00592-024-02275-4.

## Introduction

Patients with autoimmune diabetes mellitus need a lifetime replacement with exogenous insulin, and the maintenance of adequate glycemic control is considered essential for limiting the risk of diabetes-related acute and chronic complications [1]. An adequate management of the disease may become very demanding, impacting relevantly the daily routine and affecting the quality of life [2].

The clinical, social and economic burden of anxiety and depression in the general population are well known [3]. Some works have tried to delineate the prevalence of such disorders in patients with diabetes and to determine the associated factors, reporting a variable prevalence of anxiety (range 13–38%) and depression (range 9–54%) in patients with diabetes, which have been associated inconsistently with female sex, low socioeconomic status, high HbA1c, glycemic instability, risk of complications, low treatment satisfaction and worse quality of life [4–14]. However, the results of those studies are heterogeneous, due to dissimilar study design, different tools for assessing anxiety and depression, size and type of the populations under investigation [15].

The aims of our study were to investigate the prevalence of anxiety and depression in a large cohort of adult patients with autoimmune diabetes, and to explore the differences in associated factors according to sex.

## Methods

### Patients

We enrolled consecutive adult patients (age ≥ 18 years) with either Type 1 diabetes mellitus (T1DM) or latent autoimmune diabetes in adults (LADA) treated with insulin who came to the Endocrinology and Diabetes Prevention and Care Unit of the IRCCS Azienda Ospedaliera Universitaria S.Orsola-Malphighi Polyclinic of Bologna (Italy) from 04.14.2021 to 05.14.2021 to receive the second dose of SARS-CoV-2 vaccine.

The study was approved by the ethical committee of Area Vasta Emilia Centro (CE-AVEC, protocol REDIA).

### Study protocol

We administered a validated Italian translation of the following questionnaires to all patients: “*Hospital Anxiety and Depression Scale*” (HADS), “*Diabetes Distress Scale*” (DDS), “*Diabetes treatment satisfaction questionnaire*” (DTSQ) and “*Diabetes-related Quality of Life*” (DQoL).

HADS is a 14 items scale with 7 items that relate to anxiety and 7 to depression. Each item is scored from 0 to 3 so that a person can score between 0 and 21 for either anxiety or depression.

A score between 0 and 7 defines a normal condition, a score between 8 and 10 a borderline condition and a score between 11 and 21 is positive for either anxiety or depression [16].

DDS is a 17 items scale divided into four parts, with 5 items relating to “emotional burden” (feeling overwhelmed and fearful about managing the demands of diabetes over time), 5 items relating to “regimen distress” (feeling to fail by not managing diabetes well), 3 items to “interpersonal distress” (feeling to not receive sufficient support from family and friends) and 4 to “physician distress” (worrying about not receiving sufficient expertise from the health-care provider). Each item is scored from 1 to 6 and an average score of 2 to 3 in each part reflects a moderate distress, whilst a score higher or equal to 3 defines a severe distress [17].

DTSQ, originally developed by Bradley in 1990s, is composed of eight questions each of which is scored from 0 to 6: the higher the sum of all the scores, the higher the treatment satisfaction [18].

DQoL is a 46 items scale. Each item is scored from 1, labeled as “very satisfied,” to 5, labeled as “very dissatisfied.” In this case, the higher the sum of all the scores, the lower the patient’s quality of life [19].

We also collected clinical and biochemical data, data on the treatment regimen and glucose monitoring in a time span of ± 4 months from the questionnaires’ administration.

We additionally calculated the mean and the standard deviation of all HbA1c measurements during the past 3 years in all patients having at least two HbA1c measurements at intervals of 6 ± 4 months (*n* = 427; 7 patients excluded).

For individuals under intermittently scanned continuous glucose monitoring (isCGM; Free Style Libre, Abbott) or real time continuous glucose monitoring (rtCGM), we retrieved 14 days glucose metrics in patients with sensor use of > 70%. We included patients using any CGM sensor, with or without insulin pump pairing. Raw glucose data were downloaded from the system-specific web service in the form of a spreadsheet for each patient. The CGM metrics were calculated as previously described [20–22].

### Patients’ selection

From the initial cohort of 553 patients recruited, we excluded 70 subjects without HbA1c measurement ± 4 months respect to when the questionnaires were administered and 49 who did not fill in the HADS questionnaire correctly. The cohort included in the final analysis was made by 434 participants. Considering the main focus of the study on anxiety and depression, we did not exclude patients who completed the HADS but not one or more of the other questionnaires. Indeed, DDS was completed by 409 patients, DTSQ by 415 and DQoL by 303 subjects.

The patients’ selection process is shown in Supplementary Fig. 1.

### Statistical analysis

Data are shown as median and interquartile range (IQR) if not otherwise specified, or as frequencies. Continuous variables were compared by independent-samples *T* test or independent-samples Mann–Whitney *U* test as appropriate, and categorical variables were compared by *χ*^2^ test. Adjustment for multiple comparison was performed by Bonferroni method. We performed univariate analysis to address the factors associated with anxiety and depression, respectively, and to select the variables for the logistic regression model. We therefore performed logistic regression models by using backward stepwise elimination to assess the independent contributing factors associated with depression and anxiety. The model was built using the following criteria for backward stepwise elimination: 100 maximum iterations, 5 maximum step-halving, 10^–6^ parameter convergence, delta of 0, 10^–8^ as singularity tolerance. As for the stepwise options, the entry probability was 0.05, with a removal probability of 0.1 and the likelihood ratio as entry/removal test. In the model for depression, we included age, HbA1c level, sex, complications of diabetes (retinopathy/nephropathy/cardiovascular events vs. no complications), psychological disorders diagnosed by a specialist (presence vs. absence), DTSQ score, mean emotional burden DDS, mean interpersonal distress DDS and mean regimen distress DDS, whereas in that for anxiety we included HbA1c level, sex, psychological disorders diagnosed by a specialist (presence vs. absence), type of glucose monitoring (self-monitoring blood glucose, SMBG vs. CGM), DTSQ score, mean emotional burden DDS, mean interpersonal distress DDS and mean regimen distress DDS. Interaction between sex and each variable in the model was also included. Odds ratio (OR) were computed with 95% confidence interval (95%CI). Statistical analysis was performed using SPSS V.26. *p* values < 0.05 were considered significant.

## Results

Anthropometric, clinical and biochemical characteristics of the population are shown in Table 1. The majority of patients of our cohort of 434 subjects had T1DM (94%), the remaining 6% LADA. Twenty-six subjects already had a diagnosis of psychiatric/psychological disorder certified by a specialist, classified as follows: panic disorder (*n* = 2), anxiety/depression (*n* = 20), eating disorder (*n* = 2), other (*n* = 2). Among those, 14/26 (54%) were prescribed specific treatment.

**Table 1:** Anthropometric, clinical and laboratory characteristics of the whole study group

Total, *n* (%)		434 (100)
Females, *n* (%)		202 (46.5)
Age (years)		49 (33–59)
BMI (kg/m^2^)		24.2 (22.1–26.8)
Type of diabetes, *n* (%)	Type 1*	408 (94)
	LADA	26 (6)
Duration of diabetes (years)		22 (13–31)
Type of insulin therapy, *n* (%)	MDI**	363 (83.6)
	CSII	71 (16.4)
Type of glucose monitoring, *n* (%)	SMBG	155 (35.7)
	CGM***	279 (64.3)
Smoke, *n* (%)	No	251 (57.8)
	Yes	94 (21.7)
	Past	89 (20.5)
Waist circumference (cm)		87 (82–97)
HbA1c (mmol/mol)		57 (50–64)
Creatinine (mg/dL)		0.81 (0.71–0.91)
Glomerular filtration rate (ml/min)		95.6 (83.2–108.7)
LDL cholesterol (mg/dL)		102.8 (89.0–122.2)
GOT (U/L)		22 (18–26)
GPT (U/L)		19 (15–26)
Microalbuminuria (mg/L)		5 (5–8)
TSH (mcU/mL)		1.92 (1.32–3.11)
Diabetic nephropathy, *n* (%)****		12 (2.8)
Diabetic retinopathy, *n* (%)*****		142 (32.7)
Cardiovascular diseases, *n* (%)		18 (4.1)
Arterial hypertension, *n* (%)		97 (22.4)
Thyroid disease, *n* (%)		136 (31.3)
Autoimmune thyroid diseases, *n* (%)		110 (25.3)
Coeliac disease, *n* (%)		27 (6.2)
Total autoimmune diseases, *n* (%)		143 (32.9)
Neoplasms, *n* (%)		15 (3.5)
Certified psychiatric diseases, *n* (%)		26 (6)

Data are expressed as median with interquartile range in parentheses, or as frequencies

* In 184 subjects (42.4%), diabetes was diagnosed prior to the age of 18 years **basal: 13 (3%); basal-bolus: 350 (80.6%); *** isCGM: 203 (46.8%); csCGM: 76 (17.5%) ****nonproliferative diabetic retinopathy was diagnosed in 130 subjects, whereas proliferative diabetic retinopathy was diagnosed in the remaining 12 subjects *****among the 12 patients with nephropathy, 8 had proteinuria, 5 of which had microalbuminuria

*LADA* latent autoimmune diabetes of the adult, *MDI* multiple daily injections, *CSII* continuous subcutaneous insulin infusion, *SMBG* self-monitoring of blood glucose, *isCGM* intermittently scanned continuous glucose monitoring (device), *rtCGM* real-time continuous glucose monitoring (device), *BMI* body mass index, *GOT/GPT* glutamyl oxaloacetic/glutamyl pyruvic aminotransferase, *HbA1c* glycated hemoglobin, *TSH* thyroid-stimulating hormone

The results of the HADS questionnaire showed that 132 subjects (30.4%) had scores indicative of anxiety, of whom 75 subjects (17.3%) with borderline-positive scores and 57 patients (13.1%) with positive scores. The prevalence of depression was 10.8% (*n* = 47), with 7.4% (*n* = 32) having borderline-positive values and 3.5% (*n* = 15) frankly positive test.

Of note, 33/434 individuals (7.6%) scored borderline or positive for both anxiety and depression. Patients with borderline and frankly positive scores for either anxiety or depression were considered as a whole for further analyses, according to previous works [8, 23].

Supplementary Table 1 summarizes the analysis performed in the whole population stratified by HADS results. No significant difference in prevalence of anxiety and depression was observed between T1DM and LADA. In patients with anxiety, we found a higher prevalence of females (59.1% vs. 41.1%, *p* = 0.001) and of glucose-sensor users (71.2% vs. 61.3%, *p* = 0.046), as well as higher levels of HbA1c (mmol/mol: 59 [IQR 52–66] vs. 56 [IQR 49–63], *p* = 0.006), when compared to those with normal HADS score results. Patients with HADS scores indicative of depression were older and had a higher prevalence of cardiovascular diseases (12.8% vs. 3.1%, *p* = 0.002) and diabetic retinopathy (46.8% vs. 31%, *p* = 0.029), than subjects without depression.

No significant differences were detected in the prevalence of anxiety and depression among treatment groups (MDI + SMBG vs. MDI + isCGM or rtCGM vs. CSII + SMBG vs. CSII with Hybrid Closed Loop vs. CSII with Advanced Hybrid Closed Loop; *p* = 0.175 for depression and *p* = 0.246 for anxiety). The results of DDS, DTSQ and DQOL showed that patients with HADS score indicative of anxiety or depression had higher distress related to diabetes, lower treatment satisfaction and worse quality of life than those with normal HADS results (*p* < 0.001 for all comparisons), as shown in Supplementary Table 1.

Considering the relevance of the analysis of psychometric measurements according to sex, we analyzed the data separately in males and females [24]. The analysis by sex is shown in Table 2. Of note, among patients with anxiety, women showed a higher prevalence of CGM use (74.4% vs. 57.3%, *p* = 0.014), whereas men showed higher HbA1c (60 mmol/mol [IQR 52–66] vs. 55 [IQR 47–61], *p* = 0.007).

**Table 2:** Anthropometric, clinical, biochemical and psychometric parameters in patients analyzed by sex and by results of HADS questionnaire for anxiety and depression

		Anxiety						Depression					
		Females		*p* value	Males		*p* value	Females		*p* value	Males		*p* value
		HADS < 7 (*n* = 124)	HADS ≥ 8 (*n* = 78)		HADS < 7 (*n* = 176)	HADS ≥ 8 (*n* = 26)		HADS < 7 (*n* = 178)	HADS ≥ 8 (*n* = 54)		HADS < 7 (*n* = 211)	HADS ≥ 8 (*n* = 21)	
Age (years)		49 (31–61)	49 (31–55)	0.631	48 (36–59)	50 (32–56)	0.526	**46 (31–59)**	**53 (44–72)**	**0.009**	48 (34–59)	52 (40–58)	0.441
BMI (kg/m^2^)		23.2 (21.8–26.8)	23.45 (21.1–26.7)	0.616	24.7 (22.8–26.8)	24.6 (22.4–26.6)	0.470	23.2 (21.4–26.6)	24.6 (22.5–28.7)	0.074	24.7 (22.7–26.8)	24.3 (23.1–26.5)	0,.846
Type of diabetes, *n* (%)	Type 1	115 (92.7)	74 (94.9)	0.548	169 (94.9)	50 (92.6)	0.511	166 (94.3)	23 (88.5)	0.256	200 (94.8)	19 (90.5)	0.413
	LADA	9 (7.3)	4 (5.1)		9 (5.1)	4 (7.4)		10 (5.7)	3 (11.5)		11 (5.2)	2 (9.5)	
Duration of diabetes (years)		23 (14–31)	20 (12–28)	0.247	22 (13–32)	20 (12–35)	0.552	22 (13–31)	26 (16–38)	0.249	22 (13–32)	20 (12–35)	0.948
Type of insulin therapy, *n* (%)	MDI	103 (83.1)	64 (82.1)	0.853	150 (84.3)	46 (85.2)	0.871	145 (82.4)	22 (84.6)	0.779	179 (84.8)	17 (81.0)	0.639
	CSII	21 (16.9)	14 (17.9)		28 (15.7)	8 (14.8)		31 (17.6)	4 (15.4)		32 (15.2)	4 (19.0)	
Type of glucose monitoring, *n* (%)	SMBG	**53 (42.7)**	**20 (25.6)**	**0.014**	64 (35.6)	18 (33.3)	0.724	64 (36.4)	9 (34.6)	0.862	78 (37.0)	4 (19.0)	0.101
	CGM	**71 (57.3)**	**58 (74.4)**		114 (64)	36 (66.7)		112 (63.6)	17 (65.4)		133 (63.0)	17 (81.0)	
Smoke, *n* (%)	No	79 (63.7)	48 (61.5)	0.851	113 (64.2)	14 (53.8)	0.572	97 (54.5)	27 (50)	0.824	111 (52.6)	13 (61.9)	0.718
	Yes	23 (18.5)	17 (21.8)		34 (19.3)	6 (23.1)		41 (23)	13 (24.1)		50 (23.7)	4 (19.0)	
	Past	22 (17.7)	13 (16.7)		29 (16.5)	6 (23.1)		40 (22.5)	14 (25.9)		50 (23.7)	4 (19.0)	
HbA1c (mmol/mol)		58 (50–65)	59 (52–66)	0.609	**55 (47–61)**	**60 (52–66)**	**0.007**	59 (51–66)	59 (52–66)	0.977	**55 (47–61)**	**63 (52–73)**	**0.014**
Mean HbA1c 3 years before (mmol/mol)		58 (52–66)	59 (54–66)	0.292	**55 (49–61)**	**58 (53–65)**	**0.009**	58 (54–66)	60 (54–67)	0.726	56 (50–62)	59 (52–72)	0.114
SD HbA1c 3 years before		4.75 (3.17–6.39)	4.92 (3.64–7.27)	0.475	**3.82 (2.79–5.15)**	**4.42 (3.58–5.97)**	**0.038**	4.90 (3.27–6.79)	4.31 (2.81–5.67)	0.305	**3.89 (2.83–5.22)**	**4.36 (3.67–8.52)**	**0.031**
HbA1c > 53 mmol/mol, *n* (%)		87 (70.2%)	56 (71.8%)	0.804	106 (59.6)	39 (72.2)	0.092	125 (71)	18 (69.2%)	0.851	130 (61.6)	15 (71.4)	0.376
HbA1c > 48 mmol/mol, *n* (%)		103 (83.1%)	70 (89.7%)	0.187	**129 (72.5)**	**49 (90.7)**	**0.005**	149 (84.7%)	24 (92.3%)	0.299	**157 (74.4)**	**21 (100)**	**0.008**
Diabetic nephropathy, *n* (%)		3 (2.4)	1 (1.3)	0.572	5 (2.8)	3 (5.6)	0.333	4 (2.3)	0 (0.0)	0.437	7 (3.3)	1 (4.8)	0.729
Diabetic retinopathy, *n* (%)		44 (35.5)	21 (26.9)	0.205	56 (31.5)	21 (38.9)	0.310	53 (30.1)	12 (46.2)	0.102	67 (31.8)	10 (47.6)	0.141
Cardiovascular diseases, *n* (%)		4 (3.2)	4 (5.1)	0.500	7 (3.9)	3 (5.6)	0.607	**4 (2.3)**	**4 (15.4)**	**0.001**	8 (3.8)	2 (9.5)	0.217
Arterial hypertension, *n* (%)		26 (21)	13 (16.7)	0.451	44 (24.7)	14 (25.9)	0.858	32 (18.2)	7 (26.9)	0.292	53 (25.1)	5 (23.8)	0.895
Thyroid disease, *n* (%)		62 (50)	36 (46.2)	0.594	**35 (19.7)**	**3 (5.6)**	**0.014**	85 (48.3)	13 (50)	0.871	34 (16.1)	4 (19)	0.729
Autoimmune thyroid diseases, *n* (%)		51 (41.1)	30 (38.5)	0.706	**28 (15.7)**	**1 (1.9)**	**0.007**	71 (40.3)	10 (38.5)	0.855	27 (12.8)	2 (9.5)	0.665
Coeliac disease, *n* (%)		8 (6.5)	6 (7.7)	0.735	8 (4.5)	5 (9.3)	0.182	12 (6.8)	2 (7.7)	0.870	11 (5.2)	2 (9.5)	0.413
Other immune diseases, *n* (%)		57 (46)	34 (43.6)	0.741	42 (23.6)	10 (18.5)	0.433	80 (45.5)	11 (42.3)	0.763	47 (22.3)	5 (23.8)	0.872
Neoplasms, *n* (%)		7 (5.6)	3 (3.8)	0.566	3 (1.7)	2 (3.7)	0.371	8 (4.5)	2 (7.7)	0.490	4 (1.9)	1 (4.8)	0.388
Certified psychiatric diseases, *n* (%)		8 (6.5)	10 (12.8)	0.122	4 (2.2)	4 (7.4)	0.069	14 (8)	4 (15.4)	0.215	6 (2.8)	2 (9.5)	0.110
DDS* + , *n* (%)		**37 (31.4)**	**40 (56.3)**	**0.001**	**50 (29.1)**	**28 (58.3)**	** < 0.001**	**61 (36.5)**	**16 (72.7)**	**0.001**	**66 (33)**	**12 (60)**	**0.016**
emotional burden + , *n* (%)		**45 (38.1)**	**55 (77.5)**	** < 0.001**	**62 (36)**	**33 (68.8)**	** < 0.001**	**84 (50.3)**	**16 (72.7)**	**0.048**	**81 (40.5)**	**14 (70)**	**0.011**
regimen distress + , *n* (%)		**31 (26.3)**	**36 (50.7)**	**0.001**	**39 (22.7)**	**28 (58.3)**	** < 0.001**	**54 (32.3)**	**13 (59.1)**	**0.014**	**57 (28.5)**	**10 (50)**	**0.046**
interpersonal distress + , *n* (%)		**27 (22.9)**	**34 (47.9)**	** < 0.001**	**26 (15.1)**	**18 (37.5)**	**0.001**	**48 (28.7)**	**13 (59.1)**	**0.004**	38 (19)	6 (30)	0.241
Physician distress + , *n* (%)		31 (26.3)	24 (33.8)	0.270	**33 (19.2)**	**16 (33.3)**	**0.037**	46 (27.5)	9 (40.9)	0.195	45 (22.5)	4 (20)	0.798
DTSQ** (points)		**31 (27–33)**	**28 (23–31)**	** < 0.001**	**30 (26–34)**	**28 (26–32)**	**0.023**	30 (26–32)	27 (22–32)	0.069	**30 (26–34)**	**26 (25–30)**	**0.031**
DQoL*** (points)		**1.70 (1.53–1.91)**	**1.97 1.87–2.30)**	** < 0.001**	**1.62 (1.47–1.82)**	**2.04 (1.87–2.27)**	** < 0.001**	**1.76 (1.58–1.98)**	**2.21 (1.90–2.36)**	** < 0.001**	**1.64 (1.51–1.91)**	**2.00 (1.86–2.52)**	**0.001**

*Females, *n* = 189; males, *n* = 220 **females, *n* = 194; males, *n* = 221***females, *n* = 136; males, *n* = 167

Data are expressed as median with interquartile range in parentheses, or as frequencies

Significant differences are highlighted in bold

*LADA* latent autoimmune diabetes of the adult, *MDI* multiple daily injections, *CSII* continuous subcutaneous insulin infusion, *SMBG* self-monitoring of blood glucose, *isCGM* intermittently scanned continuous glucose monitoring (device), *rtCGM* real-time continuous glucose monitoring (device), *BMI* body mass index, *HbA1c* glycated hemoglobin, *SD* standard deviation

When compared to those without depression, women with depression were older (53 years [IQR 44–72] vs. 46 [IQR 31–59], *p* = 0.009) and showed a higher rate of cardiovascular diseases (15.4% vs. 2.3%, *p* = 0.001), whereas men with depression had higher HbA1c (63 mmol/mol [IQR 52–73] vs. 55 [IQR 47–61], *p* = 0.014).

Higher distress, lower treatment satisfaction and worse quality of life were detected in patients with anxiety and depression, when compared to those without, irrespectively of sex.

We then analyzed the potential factors independently associated with anxiety and depression in the entire study cohort and according to sex (Supplementary Table 2; Table 3). Risk factors for anxiety were female sex (OR 0.51, 95% 0.31–0.81; *p* = 0.005), presence of psychopathologies (OR 2.81, 95% CI 1.14–6.90; *p* = 0.024), lower DTSQ scores (OR 0.94 per 1 point increase, 95% CI 0.90–0.99; *p* = 0.011) and higher “emotional burden” DDS scores (OR 1.76 per 1 point increase, 95% CI 1.43–2.18; *p* < 0.001). No independent association between anxiety and specific variables was found according to sex. Independent risk factors for depression were increasing age (OR 1.04 per 1 year increase, 95% CI 1.01–1.06; *p* = 0.015), lower DTSQ scores (OR 0.94 per 1 point increase, 95% CI 0.88–1.00; 0.037), higher “emotional burden” DDS scores (OR 2.59 per 1 point increase, 95% CI 1.71–3.92; *p* < 0.001) and lower “regimen distress” DDS scores (OR 0.60 per 1 point increase, 95% CI 0.38–0.94; *p* = 0.027). In women, depression was independently associated with increasing age (males vs. females OR 0.96 per 1 year increase, 95% CI 0.92–1.00; *p* = 0.036). In men, depression was associated with higher HbA1c (males vs. females OR 1.08 per 1 mmol/mol increase, 95% CI 1.03–1.13; *p* = 0.002) and lower “regimen distress” DDS scores (males vs. females OR 0.40 per 1 point increase, 95% CI 0.20–0.79; *p* = 0.008).

**Table 3:** Independent risk factors for depression and anxiety in the backward stepwise model of logistic regression

Model 1: depression	OR (95% CI)	*p* value
Psychological/psychiatric diseases (presence vs. absence)	3.32 (1.00–11.05)	0.051
Age (1 year increase)	**1.04 (1.01–1.06)**	**0.015**
Sex (males vs. females)* HbA1c (1 mmol/mol increase)	**1.08 (1.03–1.13)**	**0.002**
Sex (males vs. females)* Age (1 year increase)	**0.96 (0.92–1.00)**	**0.036**
DTSQ score (1 point increase)	**0.94 (0.88–1.00)**	**0.037**
Mean DDS emotional burden (1 point increase)	**2.59 (1.71–3.92)**	** < 0.001**
Mean DDS regimen distress (1 point increase)	**0.60 (0.38–0.94)**	**0.027**
Sex (males vs females)* Mean DDS regimen distress (1 point increase)	**0.40 (0.20–0.79)**	**0.008**
Model 2: anxiety		
Sex (males vs. females)	**0.51 (0.31–0.81)**	**0.005**
Psychological/psychiatric diseases (presence vs. absence)	**2.81 (1.14–6.90)**	**0.024**
DTSQ score (1 point increase)	**0.94 (0.90–0.99)**	**0.011**
Mean DDS emotional burden (1 point increase)	**1.76 (1.43–2.18)**	** < 0.001**

Results of the logistic regression models for depression and anxiety, by using the backward stepwise elimination method. Variables included in both models were selected after univariate analysis. Variables included in the model for depression: age, HbA1c level, sex (males vs. females), complications of diabetes (retinopathy/nephropathy/cardiovascular events vs no complications), psychological disorders (presence vs. absence), DTSQ score, mean emotional burden DDS, mean interpersonal distress DDS and mean regimen distress DDS. Variables included in the model for anxiety: HbA1c level, sex (males vs. females), psychological disorders (presence vs. absence), type of glucose monitoring (CGM vs. SMBG), DTSQ score, mean emotional burden DDS, mean interpersonal distress DDS and mean regimen distress DDS. Interaction between sex and each variable in the model was also included

Significant differences are highlighted in bold

*OR* odds ratio, *CI* confidence interval, *HbA1c* glycated hemoglobin, *CGM* continuous glucose monitoring, *SMBG* self-monitoring blood glucose, *DTSQ* Diabetes Treatment Satisfaction Questionnaire, *DDS* Diabetes Distress Scale

We finally performed the analysis of the 14-days CGM indices for the subjects with available data (*n* = 122). No statistically significant difference was observed between subjects with anxiety or depression and those without, even when stratified by sex, with the exception of Time in Range (TIR) which was significantly lower in patients with anxiety (Supplementary Table 3).

## Discussion

**Fig. 1:**
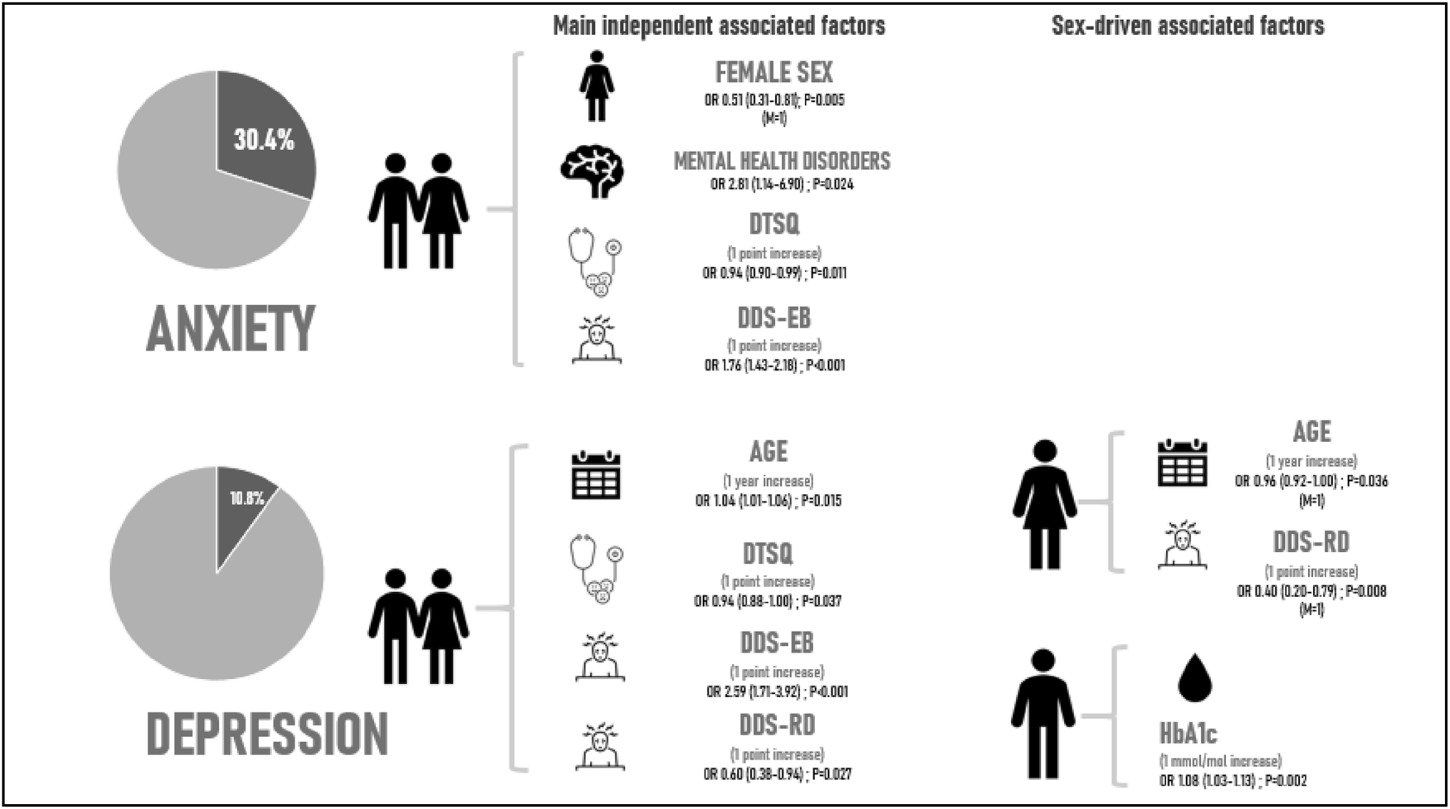
Summary of the main results on the prevalence of anxiety and depression and their independent risk factors. OR, odds ratio; DTSQ: Diabetes Treatment Satisfaction Questionnaire; DDS: Diabetes Distress Scale; EB: Emotional Burden; RD: Regimen Distress; HbA1c, glycated hemoglobin; M = males

Our study described a relevant prevalence of self-reported symptoms indicative of anxiety (30.4%) and depression (10.8%) among young and middle-aged adults with autoimmune diabetes, in the largest study so far analyzing sex-driven associated factors. A summary of the main results described above is depicted in Fig. 1.

In the clinical setting, the need to efficiently screen patients at risk of having or developing anxiety and depression has led to the creation of many dedicated questionnaires, of whom HADS has been validated and widely used [25, 26]. The prevalence of anxiety in our patients with autoimmune diabetes was two-fold higher than that of the general population assessed by HADS questionnaire, in which this condition has been reported in 15–20% of the subjects [27, 28]. The prevalence of depression seems to be in line with that reported for the general population (7–10%) [27, 28].

When focusing on adult patients with T1DM, previous studies using HADS as a screening tool have shown a prevalence of anxiety and depression of 25–38% and 8–21%, respectively [8, 9, 13, 23, 29–32]. These results were similar to those reported in these studies, which were conducted on average on younger populations of adults (range of mean age 34–45 years) with a worse metabolic control of diabetes (range of mean HbA1c 62–73 mmol/mol) than ours, indicating that anxiety and depression might be common features of patients with T1DM, relatively independent from age and metabolic control.

Remarkably, we identified a large discrepancy between individuals with a positive HADS score for anxiety and depression (*n* = 179/434, 41%) than those with a certified diagnosis of anxiety/depression (*n* = 20/434, 5%). Previous studies showed that HADS had good sensitivity and specificity, when compared to diagnoses made by semi-structured interviews for detecting depression, anxiety and panic disorders, in primary care patients and general population [33, 34]. In those settings, positive predictive values have been estimated between 30 and 50% for identification of psychiatric disorders [35, 36]. Therefore, considering our population, it may be estimated that an additional 20–40% of patients with positive HADS scores may have a clinically relevant undiagnosed psychiatric disorder. This holds true also for other disturbances, like eating disorders, which may have been likely underestimated.

When considering the whole population, subjects with anxiety had chronically worse glycemic control, as expressed by higher levels of latest HbA1c and mean HbA1c of the previous three years, and were more common users of CGM devices.

The latter evidence could have multiple explanations, since anxious subjects could prefer a real-time monitoring over SMBG, especially when fearing hypoglycemia, but for some, the great amount of data deriving from a regular use of such devices, not always easily interpretable and manageable, could feed distress and anxiety instead [37, 38]. Almost all works on CGM use and anxiety/depression have focused on pediatric patients and their caregivers, differently from ours. A recent meta-analysis on adults showed that CGM use is associated with less hypoglycemia fear, but had no impact on DTSQ score [39]. A randomized trial demonstrated that CGM use could reduce diabetic distress evaluated with DDS questionnaire [40].

Subjects with depression were older and had higher prevalence of diabetic retinopathy and cardiovascular diseases, as already documented by other works [13, 23]. Only age emerged as an independent risk factor, suggesting that the prevalence of depression increases over time in patients with autoimmune diabetes, relatively independent from potential modifiable factors.

We confirmed a higher level of distress in adults with anxiety and depression, as well as lower treatment satisfaction and worse quality of life, irrespective of sex [41, 42]. According to our results, the intrinsic emotional burden of living with diabetes and the perception of receiving inadequate insulin treatment were strong independent risk factors for both anxiety and depression irrespective of sex. Conversely, we showed no association between a higher “physician distress” and anxiety or depression prevalence, potentially resizing the role of healthcare providers regarding diabetes know-how, empathy with patients and frequency of ambulatory controls.

The analysis by sex showed specific risk factors for depression but not for anxiety. In women, age was the only independent associated factor, with a risk increment of 4% every year, differently from Melin and colleagues who described an association between depression and elevated HbA1c in women [23], so that depression seems to be related to the aging of female patients, irrespective of the metabolic control of the disease.

We highlighted a different scenario in males, where the prevalence of depression increases with increasing levels of HbA1c, with a risk of 1.08 for each 1 mmol/mol increase in HbA1c. When analyzing the data during the previous 3 years, males with anxiety and depression showed a higher SD of the HbA1c, suggesting a larger variability in the metabolic control of the disease, whereas only males with anxiety had also chronically elevated glycemia, as suggested by higher mean HbA1c of the previous 3 years. Taken all together, the data on males claim for a tighter relationship between anxiety, depression and blood glucose control, which seems to be relatively independent from other potentially modifiable parameters.

The main limitation of the study is the time of collection of questionnaires, during the COVID19 pandemic and during the vaccination, and the lack of a control group of non-diabetic individuals. An increased incidence of depression and anxiety was described during those months, especially among women [43], which could depend on many factors, such as complete or partial limitations of personal freedom, concerns for personal health, the health of loved ones and vaccine’s safety. Therefore, we cannot exclude that some of the patients with a positive questionnaire, without previously diagnosed psychopathologies, developed anxiety or depression during the pandemic, because of the cross sectional design of our study. However, the prevalence of anxiety and depression in our study fell in the lower half of the range described in the other works, which were conducted before the SARS-Cov2 pandemic. Nevertheless, the impact of the pandemic on the results of the questionnaires should be investigated in targeted studies with longitudinal data.

Another limitation is the use of a self-reported method to evaluate anxiety and depression. However, HADS has been extensively validated in the general non-diabetic population, and it is a simple and fast tool to assess these psychopathologies, which is advantageous for large populations like ours, as well as easily transferrable to clinical practice.

The strengths of our study are the large cohort involved (at the time of writing, one of the largest populations worldwide, in the literature), the completeness of clinical and biochemical data and the thorough analysis of data separately by sex.

The evidence provided by this study could impact the clinical practice but needs further investigations outside of the peculiar circumstance in which data were collected. Tracking down individuals with higher emotional burden related to diabetes, in order to offer them proper help and increasing diabetes treatment satisfaction could play a great pivotal on anxiety and depression prevention. Moreover, although with the aforementioned limits, we can hypothesize that an optimization of glycemic control could help to reduce the risk of depression in adult males with autoimmune diabetes, but not in females, where such risk would increase with aging so that the younger adults could benefit less from a psychological counseling.

In conclusion, we are progressively moving to a stage where physicians need to focus their attention toward patients’ psychological support for ultimately improving their quality of life and the metabolic control of diabetes [29, 44]. Our study highlights the sex-related differences in patients with anxiety and depression and points toward the need of fast and reliable tools to identify patients at risk who could receive a tailored treatment approach.

## Supplementary Information

Below is the link to the electronic supplementary material.

Supplementary file1 (DOCX 48 KB)
