# Supplementary material for: Sex-driven factors associated with anxiety and depression in autoimmune diabetes
Source: Acta Diabetol. 2024 May 14;61(10):1231–40. doi: 10.1007/s00592-024-02275-4 (PMC11486797; doi:10.1007/s00592-024-02275-4)
Supplement: Supplementary file 1 — Supplementary file1 (DOCX 48 KB) [file 592_2024_2275_MOESM1_ESM.docx]

Supplementary Table 1. General, anthropometric, and biochemical parameters according to the results of HADS questionnaire for anxiety and depression.

| ANXIETY | | | | | DEPRESSION | | |
| --- | --- | --- | --- | --- | --- | --- | --- |
|  | | HADS <7 (n=302) | HADS ≥8 (n=132) | p value | HADS <7 (n=387) | HADS ≥8 (n=47) | p value |
| Sex | Males, n (%) | **178 (58.9)** | **54 (40.9)** | **0.001** | 211 (54.5) | 21 (44.7) | 0.202 |
|  | Females, n (%) | **124 (41.1)** | **78 (59.1)** |  | 176 (45.5) | 26 (55.3) |  |
| Age (years) | | 48 (34-59) | 49  (32-56) | 0.418 | **47 (33-59)** | **53 (42-59)** | **0.015** |
| BMI (kg/m2) | | 24.30  (22.40-26.80) | 23.90  (21.85-26.60) | 0.209 | 24.20  (22-26.60) | 24.30  (22.70-27.50) | 0.154 |
| Type of Diabetes,  n (%) | Type 1 | 284 (94.0) | 124 (93.9) | 0.968 | 366 (94.6) | 42 (89.4) | 0.155 |
|  | LADA | 18 (6.0) | 8 (6.1) |  | 21 (5.4) | 5 (10.6) |  |
| Duration of Diabetes (years) | | 23 (13-32) | 20  (12-31) | 0.226 | 22 (13-31) | 23 (14-37) | 0.372 |
| Type of insulin therapy, n (%) | MDI | 253 (83.8) | 110 (83.3) | 0.909 | 324 (83.7) | 39 (83.0) | 0.897 |
|  | CSII | 49 (16.2) | 22 (16.7) |  | 63 (16.3) | 8 (17.0) |  |
| Type of glucose monitoring, n (%) | SMBG | **117 (38.7)** | **38 (28.8)** | **0.046** | 142 (36.7) | 13 (27.7) | 0.222 |
|  | CGM | **185 (61.3)** | **94 (71.2)** |  | 245 (63.3) | 34 (72.3) |  |
| Smoke, n (%) | No | 176 (58.3) | 75 (56.8) | 0.935 | 224 (57.9) | 27 (57.4) | 0.990 |
|  | Yes | 64 (21.2) | 30 (22.7) |  | 84 (21.7) | 10 (21.3) |  |
|  | Past | 62 (20.5) | 27 (20.5) |  | 79 (20.4) | 10 (21.3) |  |
| HbA1c (mmol/mol) | | **56 (49-63)** | **59**  **(52-66)** | **0.006** | 57 (50-63) | 60 (52-70) | 0.055 |
| HbA1c 3 years  befor e (mmol/mol) | | **56 (50-63)** | **59**  **(54-66)** | **0.002** | 57 (51-63) | 60 (53-69) | 0.133 |
| SD HbA1c 3 years before | | **4.06**  **(2.92-5.56)** | **4.67**  **(3.65-6.68)** | **0.013** | 4.21  (2.97-5.90) | 4.32  (3.64-6.91) | 0.325 |
| HbA1c >53  mmol/mol, n  (%) | | 193 (63.9) | 95 (72) | 0.102 | 255 (65.9) | 33 (70.2) | 0.554 |
| Hb1Ac >48  mmol/mol, n (%) | | **232 (76.8)** | **119 (90.2)** | **0.001** | **306 (79.1%)** | **45 (95.7%)** | **0.006** |
| Diabetic Nephropathy,  n (%) | | 8 (2.6) | 4 (3.0) | 0.824 | 11 (2.8) | 1 (2.1) | 0.778 |
| Diabetic Retinopahty,  n (%) | | 100 (33.1) | 42 (31.8) | 0.791 | **120 (31)** | **22 (46.8)** | **0.029** |
| Cardiovascular Diseases, n (%) | | 11 (3.6) | 7 (5.3) | 0.425 | **12 (3.1)** | **6 (12.8)** | **0.002** |
| Arterial Hypertension,  n (%) | | 70 (23.2) | 27 (20.5) | 0.531 | 85 (22) | 12 (25.5) | 0.579 |
| Thyroid Disease, n (%) | | 97 (32.1) | 39 (29.5) | 0.595 | 119 (30.7) | 17 (36.2) | 0.449 |
| Autoimmune Thyroid Diseases, n (%) | | 79 (26.2) | 31 (23.5) | 0.556 | 98 (25.3) | 12 (25.5) | 0.975 |
| Coeliac Disease, n (%) | | 16 (5.3) | 11 (8.3) | 0.228 | 23 (5.9) | 4 (8.5) | 0.491 |
| Other Immune Diseases, n (%) | | 99 (32.8) | 44 (33.3) | 0.910 | 127 (32.8) | 16 (34) | 0.866 |
| Neoplasms, n (%) | | 10 (3.3) | 5 (3.8) | 0.803 | 12 (3.1) | 3 (6.4) | 0.245 |

| Certified Psychiatric  Diseases, n (%) | | **12 (4)** | **14 (10.6)** | **0.007** | **20 (5.2)** | **6 (12.8)** | **0.038** |
| --- | --- | --- | --- | --- | --- | --- | --- |
| DDS* +, n (%) | 155 (37.9) | **87 (30)** | **68 (57.1)** | **<0.001** | **127 (34.6)** | **28 (66.7)** | **<0.001** |
| emotional burden  +, n (%) | 195 (47.7) | **107 (36.9)** | **88 (73.9)** | **<0.001** | **165 (45)** | **30 (71.4)** | **0.001** |
| regimen distress +, n (%) | 134 (32.8) | **70 (24.1)** | **64 (53.8)** | **<0.001** | **111 (30.2)** | **23 (54.8)** | **0.001** |
| interpersonal distress +, n (%) | 105 (25.7) | **53 (18.3)** | **52 (43.7)** | **<0.001** | **86 (23.4)** | **19 (45.2)** | **0.002** |
| physician distress  +, n (%) | 104 (25.4) | **64 (22.1)** | **40 (33.6)** | **0.015** | 91 (24.8) | 13 (31) | 0.385 |
| DTSQ** (points) | | **30 (26-34)** | **28 (24-32)** | **<0.001** | **30 (26-33)** | **26 (23-32)** | **0.005** |
| DQoL*** (points) | | **1.63**  **(1.51-1.84)** | **2**  **(1.87-2.27)** | **<0.001** | **1.71**  **(1.53-1.93)** | **2.02**  **(1.89-2.38)** | **<0.001** |
| *n=409, **n=415, ***n=303 | | | | | | | |
| Data are expressed as median with interquartile range in parentheses, or as frequencies.  LADA, latent autoimmune diabetes of the adult; MDI, multiple daily injections; CSII, continuous subcutaneous insulin infusion; SMPG, self-monitoring of plasma glucose; isCGM, intermittently scanned continuous glucose monitoring (device); rtCGM, real-time continuous glucose monitoring (device); BMI, body mass index; HbA1c, glycated hemoglobin; SD, standard deviation | | | | | | | |

Supplementary Table 2. CGM metrics according to the results of HADS questionnaire for anxiety and depression

| ANXIETY | | | | DEPRESSION | | |
| --- | --- | --- | --- | --- | --- | --- |
|  | HADS <7 | HADS >8 | p value | HADS <7 | HADS >8 | p value |
| Mean Glycemia  (mg/dL) | 155 (144-175) | 170 (149-195) | 0.056 | 161  (145-181) | 175 (152-205) | 0.182 |
| Median Glycemia  (mg/dL) | 148 (138-166) | 164 (140-189) | 0.054 | 154  (138-172) | 171 (144-198) | 0.196 |
| GMI (%) | 7 (6.8-7.5) | 7.4 (6.9-8) | 0.055 | 7.2 (6.8-7.7) | 7.5 (7.0-8.3) | 0.185 |
| GMI (mmol/mol) | 53 (50-58) | 57 (52-64) | 0.056 | 55 (50-60) | 59 (52-66) | 0.185 |
| SD Glycemia | 61 (49-66) | 63 (54-76) | 0.156 | 61 (50-69) | 58 (51-78) | 0.586 |
| CV (%) | 37.1 (32.3-41.5) | 37 (33.7-41.5) | 0.979 | 37.1  (33.4-41.5) | 36.6 (30.7-41.6) | 0.828 |
| TIR (%) | **60.1 (52.7-72.1)** | **54.8 (43.4-66.6)** | **0.043** | 59.7  (51.4-72.1) | 56.3 (39.6-61.2) | 0.174 |
| TBR (%) | 4 (1.2-6.7) | 2.2 (1-5.7) | 0.454 | 3.9 (1.3-6.2) | 1.4 (0.6-8.1) | 0.259 |
| TAR (%) | 32.3 (22.9-42.7) | 40.8 (26.1-54.7) | 0.061 | 36.1  (22.9-45.8) | 43.3 (30.9-59) | 0.161 |
| TBR2 (%) | 0.3 (0.1-1.3) | 0.4 (0.1-0.9) | 0.916 | 0.4 (0.1-1.1) | 0.1 (0.1-1.7) | 0.513 |
| TBR1 (%) | 3.1 (1.1-5.4) | 1.9 (1-4.4) | 0.377 | 3.1 (1.1-5) | 1.3 (0.6-6.4) | 0.259 |
| TAR1 (%) | 23.9 (18.3-28.7) | 26.8 (20.9-31.8) | 0.206 | 24.9  (18.8-28.9) | 29.3 (21.2-35.3) | 0.121 |
| TAR 2 (%) | 8.5 (2.8-14.4) | 11.2 (4.2-22.6) | 0.074 | 9.1 (3-16.5) | 9 (5.7-27.7) | 0.378 |
| Daily Scans with  isCGM, n (%) | 9 (6-13) | 6 (5-10) | 0.111 | 8 (6-13) | 9 (5-18) | 0.811 |
| Data are expressed as median with interquartile range in parentheses, or as frequencies.  GMI, glucose management indicator; SD, standard deviation; CV, variation coefficient; TIR, time in range; TBR, time below range; TAR, time above range; TBR2, time below range <55 mg/dL; TBR1, time below range 55-70 mg/dL; TAR1, time above range 180-250 mg/dL; TAR2, time above range >250 mg/dL; isCGM, intermittently scanned continuous glucose monitoring | | | | | | |

**Supplementary Table 3. Independent risk factors for depression and anxiety in the univariate analysis**

| **Model 1: depression** | **OR (95% CI)** | **P value** |
| --- | --- | --- |
| Insulin pump vs MDI |  | 0.897 |
| Autoimmune diseases (presence vs absence) |  | 0.866 |
| Psychological/psychiatric diseases (presence *vs* absence) | **2.69 (1.02-7.07)** | **0.045** |
| Glucose monitoring (CGM vs SMBG) |  | 0.255 |
| Complications related to diabetes (presence vs absence) | 1.82 (0.99-3.36) | 0.054 |
| Sex (males vs females) |  | 0.204 |
| HbA1c (1 mmol/mol increase) | 1.02 (1.00-1.05) | 0.056 |
| Age (1 year increase) | **1.03 (1.01-1.05)** | **0.013** |
| Duration of diabetes (1 year increase) |  | 0.296 |
| Insulin/kg (1 unit increase) |  | 0.165 |
| Mean HbA1c (1 mmol/mol increase) |  | 0.122 |
| SD HbA1c (1 SD increase) |  | 0.952 |
| DTSQ score (1 point increase) | **0.92 (0.88-0.97)** | **0.003** |
| Mean DDS (1 point increase) | **1.51 (1.15-1.99)** | **0.003** |
| Mean DDS emotional burden (1 point increase) | **1.81 (1.40-2.34)** | **<0.001** |
| Mean DDS regimen distress (1 point increase) | 1.30 (1.00-1.68) | 0.052 |
| Mean DDS interpersonal distress (1 point increase) | **1.47 (1.18-1.82)** | **0.001** |
| Mean DDS physician distress |  | 0.827 |
|  |  |  |
| **Model 2: anxiety** |  |  |
| Insulin pump vs MDI |  | 0.909 |
| Autoimmune diseases (presence vs absence) |  | 0.910 |
| Psychological/psychiatric diseases (presence *vs* absence) | **2.87 (1.29-6.38)** | **0.010** |
| Glucose monitoring (CGM vs SMBG) | **1.56 (1.01-2.44)** | **0.047** |
| Complications related to diabetes (presence vs absence) |  | 0.823 |
| Sex (males vs females) | **0.48 (0.32-0.73)** | **0.001** |
| HbA1c (1 mmol/mol increase) | **1.02 (1.00-1.04)** | **0.030** |
| Age (1 year increase) |  | 0.390 |
| Duration of diabetes (1 year increase) |  | 0.195 |
| Insulin/kg (1 unit increase) |  | 0.612 |
| Mean HbA1c (1 mmol/mol increase) | **1.02 (1.00-1.04)** | **0.016** |
| SD HbA1c (1 SD increase) |  | 0.130 |
| DTSQ score (1 point increase) | **0.92 (0.89-0.96)** | **<0.001** |
| Mean DDS (1 point increase) | **1.79 (1.45-2.21)** | **<0.001** |
| Mean DDS emotional burden (1 point increase) | **1.93 (1.58-2.36)** | **<0.001** |
| Mean DDS regimen distress (1 point increase) | **1.65 (1.36-2.00)** | **<0.001** |
| Mean DDS interpersonal distress (1 point increase) | **1.60 (1.35-1.91)** | **<0.001** |
| Mean DDS physician distress |  | 0.095 |
| OR: odds ratio; CI: confidence interval; MDI: multiple daily injections; HbA1c: glycated hemoglobin; CGM: continuous glucose monitoring; SMBG: self-monitoring blood glucose; DTSQ: Diabetes Treatment Satisfaction Questionnaire; DDS: Diabetes Distress Scale. | | |

Enrolled subjects (n=533)

Excluded for HADS compilation error (n=49)

Analyzed subjects (n=434)

Excluded for missing HbA1c (n=70)

**Supplementary Figure 1. Flow chart of the patients’ selection process.**
